# The economic burden of COVID-19 undervaccination: costs of hospitalisation, ICU admission, and death in Scotland

**DOI:** 10.1186/s13561-026-00775-3

**Published:** 2026-04-21

**Authors:** Steven Kerr, Joan E. Madia, Catia Nicodemo, Aziz Sheikh

**Affiliations:** 1 Centre for Medical Informatics, University of Edinburgh, Edinburgh, UK; 2 Department of Primary Care and Health Sciences, Medical School, University of Oxford, Oxford, UK

## Abstract

The COVID-19 pandemic has imposed substantial financial and operational pressures on healthcare systems globally. While vaccines were known to reduce severe outcomes, their broader economic impacts, especially in publicly funded health systems, requires clearer quantification. This study evaluates the direct healthcare costs associated with COVID-19 outcomes in relation to vaccination status, using linked individual-level data from Scotland’s EAVE II cohort (June to September 2022). We defined undervaccination as the shortfall between recommended and received COVID-19 vaccine doses, stratified by age group. Retrospective cohort analysis employing Cox proportional hazards and linear regression models was used to assess the associations between undervaccination and the risk, frequency, and duration of hospitalisation, intensive care unit (ICU) admission, and death. We also conducted a counterfactual analysis to estimate averted costs and quality-adjusted life years gained (QALYs) under a full vaccination scenario.

Findings demonstrate a strong dose–response relationship between undervaccination and severe COVID-19 outcomes. Among individuals aged 75+, those with higher undervaccination levels showed significantly increased hazard ratios for hospitalisation (up to 3.92 for sub-optimal level 2), ICU admission (up to 12.53 for sub-optimal level 2), and mortality (up to 6.63 for sub-optimal level 3). These elevated risks translated into substantial direct healthcare costs. Hospitalisation costs reached £4.7 million for the 75+ group and £2.6 million for the 16–74 group, while ICU costs totalled £70,489 and £246,486, respectively. Under a counterfactual full vaccination scenario, potentially avertable hospital costs were estimated at £1.4 million for the 75+ group and £0.5 million for the 16–74 group. Vaccination rates were lower among younger individuals, ethnic minorities, and residents in more deprived areas, highlighting persistent health inequalities. Of the total cohort of 4,992,498 individuals, 65.8% were fully vaccinated on 1 June 2022, while 34.2% were undervaccinated. These inequalities were not only clinically consequential but also economically costly: undervaccinated individuals generated more than five-fold the COVID-19 healthcare spend of their fully vaccinated peers. Universal full vaccination could have averted approximately £1.4 million (75+) and £0.5 million (16–74) in hospital costs, and a further £38,000 in ICU costs, during summer 2022.

**Supplementary Information:**

The online version contains supplementary material available at 10.1186/s13561-026-00775-3.

## Introduction

The COVID-19 pandemic has exerted sustained pressure on healthcare systems worldwide, precipitating surges in hospitalisations, increased demand for intensive care, and considerable economic strain [1–3]. In Scotland alone, the Government earmarked £2.9 billion for pandemic-related health costs in 2020/21, with NHS National Services Scotland projecting a further £399 million in 2021/22 [1]. Across the United Kingdom, COVID-19 support measures have added an estimated £344 billion to public borrowing, including £35 billion on preventive care such as vaccination, testing and personal-protective equipment in 2021 [4]. Internationally, average health expenditure in OECD countries jumped from 8.8% of GDP in 2019 to 9.7% in 2021 [4], while in the United States widespread vaccination was estimated to have averted US$1.15 trillion in direct medical costs [3]. Equitable vaccine distribution could yield a further US$466 billion in economic gains across major economies by 2025, underscoring the broader economic value of high vaccine uptake [5].

A growing body of international evidence indicates that COVID-19 vaccination programmes generate substantial cost savings by reducing hospital admission rates, intensive care unit (ICU) usage, and treatment costs. In the United States, cost-effectiveness analyses suggest that direct healthcare savings range from $$\$602.8$$ million to $$\$3.5$$ billion, varying with assumed vaccine uptake and population risk profiles [6, 7]. One study estimated a cost per quality-adjusted life year (QALY) gained of approximately $8,200, highlighting the economic value of vaccination. di Fusco et al. [8] also found that the Pfizer-BioNTech vaccine alone prevented hundreds of thousands of hospitalisations and deaths in its first year, generating substantial public health and fiscal gains.

Canadian modelling comparing the national COVID-19 vaccination programme with a no-vaccination counterfactual estimated a net monetary benefit of CAD 298.1 billion ($\simeq$ USD 220.6 billion) over its first 16 months (14 December 2020–31 March 2022), yielding 6.61 million QALYs; complementary analyses of post-COVID condition indicate healthcare savings of CAD 1,675–7,340 (USD 1,240–5,430) per acute infection in the first post-infection year [9, 10]. Similarly, studies from Europe document robust economic returns. In the Basque Country, Spain, vaccination programmes achieved direct savings of €26.4 to €28.9 million (USD 28.5–31.2 million), along with 16% and 19.6% reductions in hospitalisations and ICU usage, respectively [11]. Comparable findings from Italy, Austria, and other high-income countries support a global consensus on the cost-effectiveness of vaccination [12, 13].

UK-specific modelling corroborates these conclusions. Sandmann et al. [14] projected that, without vaccination, COVID-19 could result in cumulative healthcare costs of £85.6 billion over a ten-year period. Harrison et al. [15] estimated reductions in hospital admissions ranging from 38% to 90%, depending on uptake and variant circulation. More recent analyses by Kohli et al. [7] on the Autumn 2024 bivalent mRNA vaccines demonstrated their cost-effectiveness, even under conservative assumptions, especially for high-risk groups.

Other studies further underscore the value of mitigation strategies. The CALMS model by Mintram et al. [16] used agent-based simulations to show the long-term benefits of sustained public health interventions in the UK. Evaluations of the 2022 and 2023 booster campaigns by Mendes et al. [17] found continued reductions in hospitalisations and mortality. A systematic review by Vardavas et al. [18] covering the European Union (EU), the United Kingdom (UK), and OECD countries reaffirmed vaccination as one of the most cost-effective public health interventions.

Despite this, estimates of vaccine-related cost impacts in the UK vary widely, influenced by differing modelling assumptions, timeframes, and definitions of direct and indirect costs. Some studies report short-term increases in NHS expenditure (£112–665 million; approximately USD 141–838 million) due to large-scale procurement and delivery efforts [7]. However, these were often offset by long-term savings from avoided healthcare utilisation.

Recent UK-wide analyses also highlight the risks of undervaccination. A meta-analysis by Kerr et al. [19], using harmonised electronic health records from across the four nations, found that undervaccinated individuals -defined as having received fewer than the recommended doses- faced significantly higher risks of hospitalisation and death. As of June 2022, rates of undervaccination ranged from about one-third to one-half of the population across the four UK nations, with the highest levels in younger, non-White, and socio-economically deprived groups. In most age groups, adjusted hazard ratios for severe outcomes tended to rise with each additional missed dose; among people aged 75+, the highest observed adjusted hazard ratio was 3.61 for those three doses short of the recommended schedule. The authors estimated that over 7,000 severe cases could have been prevented during Summer 2022 had full vaccination coverage been achieved.

In the present study, we provide a novel and more granular analysis of the direct economic impact of COVID-19 vaccination. While previous work, such as the UK-wide meta-analysis by [19] established a clear link between undervaccination and a composite outcome of hospitalisation and death, our research disaggregates these severe outcomes. The Kerr paper defined a “severe COVID-19 outcome” as “COVID-19 hospitalisation or death”. In contrast, we present separate, distinct models for COVID-19-related hospitalisation, ICU admission, and death. This approach allows for a more detailed understanding of the specific health and economic burdens associated with each outcome. Furthermore, while the previous work relied on a meta-analysis to combine data from four nations, our study utilizes detailed patient-level administrative health data from a single cohort in NHS Scotland. This approach allows us to contribute to the growing evidence base by providing a detailed assessment of the direct healthcare costs associated with COVID-19 vaccination status in Scotland during Summer 2022. Specifically, we compare hospitalisation, ICU use, and treatment-related costs between vaccinated and unvaccinated individuals, contributing to the growing evidence base with a national, population-level perspective.

More precisely, this study uses individual-level, linked health data from the EAVE II platform, which includes the entire population registered with a general practitioner in Scotland as of February 2020 (approximately 5.4 million individuals). The dataset comprises routinely collected records on COVID-19 testing, vaccination status, hospital and ICU admissions, mortality, and demographic characteristics. Our analysis focuses on the period from 1 June to 30 September 2022, a time of substantial viral circulation and NHS pressure. By leveraging this comprehensive national dataset, we were able to capture real-world healthcare utilisation and costs associated with differing levels of COVID-19 vaccination, enabling robust comparison between vaccinated and unvaccinated individuals across age, risk, and socioeconomic groups, see Kerr et al. [19] for more details.

We employed Cox proportional hazards models to estimate the association between undervaccination and the risk of hospitalisation, ICU admission, and death due to COVID-19, adjusting for key covariates including age, sex, socioeconomic status, ethnicity, and clinical risk factors. For hospital and ICU stay durations, we used linear regression models with log-transformed outcomes. An observed–expected analysis was conducted to simulate counterfactual scenarios under full vaccination coverage, estimating the number of events, bed-days, and associated costs potentially averted.

## Methods

This study used the EAVE II (Early Pandemic Evaluation and Enhanced Surveillance of COVID-19) platform, consisting of a nationally representative cohort comprising approximately 5.4 million individuals who were registered with a general practitioner (GP) in Scotland as of 23 February 2020. Based on the National Records of Scotland (NRS) 2019 mid-year population estimates, this cohort captured about 98–99% of the total Scottish population [20]. For the source of data see Table S1 in the Supplemental Material.

Primary care data formed the population spine for this dataset. These were linked with a wide range of additional data sources using the Community Health Index (CHI), a unique identifier assigned to all individuals receiving NHS services in Scotland. Hospital-related data were sourced from Scottish Morbidity Records (SMR01). Intensive care data come from the Scottish Intensive Care Society Audit Group (SICSAG). Mortality outcomes were captured using NRS death registrations. Laboratory data, including RT-PCR test results, were sourced from Electronic Communication of Surveillance in Scotland (ECOSS). Vaccine records come from the Turas Vaccine Management Tool, an online platform used by healthcare professionals in Scotland to document patient vaccination information in real time at the point of care.

The study period spanned from 1 June to 30 September 2022, and focused on evaluating the healthcare and economic impacts of COVID-19 vaccination during this time.

### Study design and population

We conducted a prospective observational cohort study of individuals aged 5–85+ who were eligible for COVID-19 vaccination in Scotland. The main analyses presented in the text focus on individuals aged 16 years and older, as those under 16 had lower vaccine requirements and experienced no ICU admissions or deaths, only hospitalisations. Results for the 5–15-year-old age group are provided in the Supplementary Materials.

Eligibility and dose schedules were determined based on national vaccination guidelines in effect prior to 1 June 2022, as summarised in Table S2 of the Supplemental Material. This date was used consistently across analyses to define undervaccination status.

According to the national schedule, individuals aged 16–74 were eligible to receive two primary vaccine doses and one booster by February 28th, 2022. Individuals aged 75 and older were eligible for two primary doses and two booster doses by March 25th, 2022. Undervaccination status was defined as having received fewer doses than the standard number offered to one’s age group by 1 June 2022. For example, individuals aged 16–74 with zero, one, or two doses, and individuals aged 75+ with zero to three doses, were classified as undervaccinated.

This definition was chosen to enable consistent comparison across UK nations and was based solely on age-specific eligibility. While some individuals may have been offered additional doses due to clinical risk factors (e.g., immunosuppression), we did not account for such exceptions due to difficulties in reliably identifying these subgroups.

To be included in the cohort, individuals were required to have sex and age (or year of birth) recorded, be alive and resident in Scotland on 1 June 2022, and have a valid identifier of residential location. This study population formed the basis for two analyses: (i) a descriptive analysis of undervaccination status as of 1 June 2022, and (ii) a follow-up study examining severe COVID-19 outcomes from June 1 st to September 30th, 2022.

### Statistical analysis

We conducted a retrospective cohort study that uses the EAVE II data platform.

To estimate the association between undervaccination and the risk of severe COVID-19 outcomes, we employed Cox proportional hazards models with time-to-event outcomes. Separate models were fitted for COVID-19-related hospitalisation, ICU admission, and death. The primary exposure variable was undervaccination (ranging from 0 to 4), defined relative to the recommended number of doses by age group as of 1 June 2022.

Models were adjusted for a comprehensive set of demographic, socioeconomic, and clinical covariates. Demographic and household variables included age group, sex, ethnicity, number of people in the household, and mean household age. Socioeconomic status was captured by the Scottish Index of Multiple Deprivation (SIMD quintile) and urban/rural classification.

Health-related covariates included the number of QCovid-defined risk groups [21], smoking status, blood pressure, and previous SARS-CoV-2 infection status. Clinical history adjustments were made for housing status (care home or homelessness), learning disability and Down’s syndrome, chronic kidney disease (CKD), and a range of pre-existing conditions including: atrial fibrillation, asthma, blood cancer, cardiac and cerebrovascular diseases (e.g. coronary heart disease, congestive heart failure, stroke), liver cirrhosis, congenital heart disease, chronic obstructive pulmonary disease (COPD), diabetes types 1 and 2, epilepsy, neurological conditions (including Parkinson’s disease), dementia, pulmonary hypertension, cystic fibrosis or bronchiectasis, venous thromboembolism, cancer (lung, oral), HIV/AIDS, severe combined immunodeficiency, sickle cell disease or other immunodeficiencies, rheumatoid arthritis or systemic lupus erythematosus, and severe mental illness. Rather than including each comorbidity individually, we derived a total count of comorbidities per individual. Missing values for smoking and blood pressure were handled by including missingness as a separate category. Although available in the dataset, BMI was excluded as a covariate because 61.1% of the cohort had missing values. BMI may be more frequently recorded by GPs when it falls outside the normal range, and as a result the data likely violate the missing-at-random (MAR) assumption. We therefore did not impute BMI, and opted to omit the variable from the final analysis.

The hazard function at time $t$ for individual $i$ was modeled as:

$$\begin{aligned} h_i(t) = h_0(t) \exp \left( \beta _1 \cdot \text {Vaccinesub-optimal}_i + \sum \limits _{k=2}^{p} \beta _k \cdot X_{ik} \right) \end{aligned}$$

where $h_0(t)$ was the baseline hazard function, $\text {Vaccinesub-optimal}_i$ was a categorical variable (0–4) representing undervaccination level, and $X_{ik}$ denotes the $k$-th covariate for individual $i$. The exponentiated coefficients $\exp (\beta _k)$ represent hazard ratios associated with each covariate.

For each outcome, fully vaccinated individuals (sub-optimal = 0) served as the reference category. Models were fitted separately for individuals aged 16–74 and those aged 75 and above, to account for differences in vaccine eligibility timelines and underlying risk profiles.

As with other EAVE II–based analyses, loss to follow-up could occur through individuals leaving Scotland, which was not directly observable in the available data; censoring was therefore assumed to be non-informative conditional on observed covariates, consistent with assumptions made in previous EAVE II studies.

### Exposure definition: undervaccination

The primary exposure variable was the *undervaccination*, defined as the number of COVID-19 vaccine doses recommended by the Joint Committee on Vaccination and Immunisation (JCVI), minus the number of doses actually received. The standard schedule used for defining full vaccination in Summer 2022 in the UK was:

- Ages 16–74: 3 doses
- Ages 75 and older: 4 doses

### Outcomes and statistical models

#### Severe COVID-19 outcomes

We fit separate Cox proportional hazards models with a time-dependent undervaccination variable as the exposure. The outcomes of interest were:

- COVID-19 hospitalisation: Emergency admissions with COVID-19 recorded as the primary cause
- COVID-19 ICU admission: ICU stays during hospitalisation episodes classified as COVID-19 admissions
- COVID-19 death: Deaths with COVID-19 listed as the primary underlying cause

Covariates in the adjusted models included sex, age group, urban/rural classification, SIMD quintile (deprivation index), ethnicity, and number of QCovid risk groups.

**Fig. 1:**
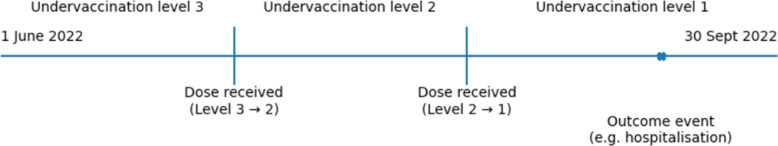
Construction of time-varying undervaccination status during follow-up

Undervaccination status was included as a time-varying exposure. When an individual’s undervaccination status changed, their follow-up was split into additional intervals in the analysis dataset, with each interval corresponding to an observation period in which the undervaccination variable remained constant. This is illustrated schematically in Fig. 1.

The risk ratio for the counterfactual scenario in which an individual’s undervaccination was zero was calculated as:

$$\begin{aligned} \exp (-\hat{\beta _1}) \end{aligned}$$

where $\hat{\beta }_1$ was the estimated regression coefficient for the observed undervaccination of the individual.

Similar models with a composite outcome of COVID-19 hospitalisation or death were fitted and reported on in Kerr et al. [19]. Separate models for hospitalisation, death and ICU admission were fitted as part of the current study.

#### Duration of hospital and ICU stay

We modelled the duration of COVID-19-related hospital and ICU stays using linear regression models with $\ln (\text {duration})$ as the dependent variable. The exposure was undervaccination at the time of admission. Adjustments included all aforementioned covariates, plus the specific location of the hospital of admission and month of admission. The regression coefficients were exponentiated and interpreted as *duration ratios*. All eligible stays that began during the study period were included, even if they extended beyond the end of follow-up.

### Observed–expected analysis

To estimate the number of events and hospital bed-days averted under a full vaccination scenario, we performed an *observed–expected* comparison. For individuals with events and an undervaccination $\ge 1$, we assumed a counterfactual event probability of $\exp (-\hat{\beta }_1)$. For durations, counterfactual predictions were based on the expected mean duration from the regression model, multiplied by the counterfactual probability of admission. Independence between probability of event and duration was assumed for variance estimation.

Vaccination status was modelled as a time-varying exposure. Undervaccination status was updated dynamically during follow-up, such that individuals contributed person-time to the undervaccination level corresponding to their vaccination status at each point in time. When an individual received an additional vaccine dose during the study period, they transitioned to a lower undervaccination level on the date of vaccination, and subsequent person-time was attributed to the updated exposure category. Cox proportional hazards models were estimated using a counting-process formulation, allowing individuals to contribute multiple observation intervals across different undervaccination levels. (Fig. 1).

### QALY gains and cost inputs

The economic analysis was conducted from the perspective of the NHS and was intentionally restricted to direct secondary-care costs associated with COVID-19-related hospitalisations and ICU admissions. Costs related to primary care, ambulance services, emergency department attendances not resulting in admission, long COVID or post-acute sequelae, social care, informal caregiving, and productivity losses were not included. This restricted scope was chosen to align with the study objective of quantifying directly observable hospital-sector costs using routinely collected administrative data. As such, all cost estimates presented in this study should be interpreted as conservative lower-bound estimates of the true economic burden of COVID-19 undervaccination.

Following the approach of Kohli et al. [7], QALYs gained were calculated from averted deaths, applying an annual discount rate of 3.5% and age-specific utility weights. We calculated the quality-equivalent cost of death (QECD), defined as the monetary valuation of mortality adjusted for remaining life expectancy and age-specific health utility weights. Morbidity-related quality-of-life losses from hospitalisation and ICU stays were not included in the base-case analysis. A supplementary sensitivity analysis exploring the potential contribution of morbidity-related QALY losses is presented in the Supplementary Materials (section 2, Tables S5–S8), demonstrating that inclusion of these losses would substantially increase the estimated health benefits of vaccination and further reinforce the conservative nature of our base-case estimates.

Vaccine costs were set at values derived from NHS pricing data [22, 23]:

- £7.54 (approx. $9.43) per administered dose (service delivery fee)
- £64 (approx. $80) per Pfizer dose
- £67 (approx. $83.75) per Moderna dose

These estimates were used in the cost-effectiveness analysis to compare the healthcare cost savings and QALYs gained against the cost of vaccine procurement and delivery.

## Results

### Participants

**Fig. 2:**
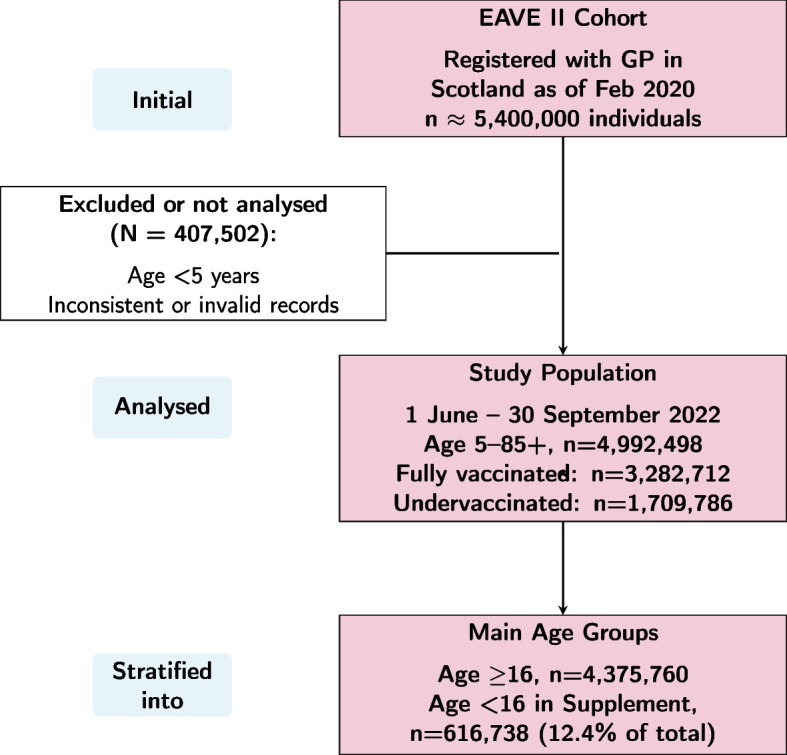
Flow diagram of the study population from EAVE II to the main analysis. The primary analysis included individuals aged 16 and over; individuals under 16 were analysed separately in the Supplementary Materials. Vaccination status is indicated for the study population

Participants were identified from the EAVE II (Early Pandemic Evaluation and Enhanced Surveillance of COVID-19) platform, a nationally representative cohort of approximately 5.4 million individuals registered with a general practitioner (GP) in Scotland as of February 2020 [20]. The study period spanned 1 June to 30 September 2022, encompassing 4,992,498 individuals aged 5–85+. The cohort was stratified by vaccination status, including 3,282,712 fully vaccinated and 1,709,786 undervaccinated individuals (Fig. 2).

For the main analyses, we focused on individuals aged 16 and older, as those under 16 had lower vaccination requirements and experienced only hospitalisations, with no ICU admissions or deaths. Results for the under-16 population are reported in the Supplementary Materials. This younger cohort included 616,738 individuals, representing approximately 12.4% of the total study population.

### Descriptive statistics

Table S3 in the Supplemental Materials presents the descriptive characteristics of the study cohort, stratified by vaccination status. The total sample includes 3,282,712 individuals who were fully vaccinated (65.8%) and 1,709,786 who were undervaccinated (34.2%). For each covariate, the table displays the count and row percentage within each vaccination group. For example, among females, 69.0% were fully vaccinated, compared to 62.5% of males.

Clear age differences were observed across groups: the mean age among fully vaccinated individuals was 51.1 years (SD: 19.9), while among the undervaccinated it was substantially lower at 30.8 years (SD: 19.0). Younger age groups, such as those aged 5–11, 12–15, and 16–17, were notably underrepresented among the fully vaccinated, reflecting age-based vaccine eligibility during the study period. Conversely, individuals aged 60 and above had high levels of full vaccination coverage, exceeding 90% in many strata.

Vaccination uptake was also patterned by socioeconomic status: in the most deprived quintile (SIMD 1), only 53.9% were fully vaccinated, while in the least deprived quintile (SIMD 5), this figure rose to 73.6%. Urban residents made up the majority of the cohort, with 63.7% of urban individuals being fully vaccinated, compared to 71.4% in rural areas.

Ethnic disparities were apparent, with White individuals showing higher vaccination uptake (69.7%) than Asian (56.8%), Black (39.0%), Mixed (43.7%), and Other ethnicities (42.5%). Individuals in larger households, particularly those with 6–10 members, were more likely to be undervaccinated (60.9%) compared to those living alone (30.7%).

Health status indicators also reveal important gradients: individuals with multiple risk conditions had substantially higher vaccination rates (e.g., 86.5% for those with three risk groups), as did those with chronic conditions such as atrial fibrillation (87.7%) or chronic kidney disease (CKD stage 3: 87.5%). However, conditions with smaller prevalence, such as HIV/AIDS or Down’s syndrome, also show high vaccine uptake, suggesting targeted efforts.

There was substantial missing data for some variables, notably body mass index (BMI), with over 60% missingness, and smoking status, where the missing category constitutes a larger proportion of the undervaccinated group (58.4%). Blood pressure records were also missing for a significant portion of the cohort, and again, missingness appears more common among the undervaccinated.

Overall, the descriptive statistics in this table suggest that demographic, socioeconomic, and clinical differences between fully vaccinated and undervaccinated individuals may influence both vaccine uptake and subsequent outcomes. Therefore, subsequent analyses adjust for these covariates to better isolate the association between vaccination status and health outcomes.

### Cox model results

Building on the descriptive statistics, we now turn to models to assess the association between undervaccination and hospitalisation risk, adjusting for relevant covariates. Tables 1 and 2 present results from Cox proportional hazards models estimating the risk of hospitalisation due to COVID-19 across different levels of undervaccination, separately for individuals aged 16–74 and those aged 75 and above. The undervaccination variable captures the cumulative number of missed vaccine doses relative to the age-specific recommended schedule, with level 0 denoting full vaccination.

**Table 1:** Cox model of risk of being hospitalised, 16–74 years old

Variable	Levels	PY (thousands)	Persons	Events	Rate (/1000 PY)	HR (95% CI)
undervaccination	0	945.157	3,104,878	1,496	1.58	Ref
	1	156.665	502,377	213	1.36	1.79 (1.54, 2.08)
	2	36.385	116,814	66	1.81	2.63 (2.04, 3.39)
	3	131.970	401,421	223	1.69	2.34 (2.02, 2.72)
R$^{2}$	0.001					
Max. R$^{2}$	0.015					
Log Likelihood	−28,624.420					
Wald Test	3,122.250$$^{***}$$ (df = 30)					
LR Test	2,967.902$$^{***}$$ (df = 30)					
Score Test	5,288.664$$^{***}$$ (df = 30)					

All models adjusted for sex, age group, urban/rural classification, SIMD quintile, ethnicity, and QCovid risk groups

*PY* Person years, *HR* Hazard ratio, *CI* Confidence interval

$$^{*}$$*p*<0.1; $$^{**}$$*p*<0.05; $$^{***}$$*p*<0.01

**Table 2:** Cox model of risk of being hospitalised, 75+ years old

Variable	Levels	PY (thousands)	Persons	Events	Rate (/1000 PY)	HR (95% CI)
undervaccination	0	130.945	590,636	1,028	7.85	Ref
	1	12.037	53,660	294	24.42	2.71 (2.37, 3.09)
	2	2.244	7,351	70	31.19	3.92 (3.07, 5.00)
	3	0.346	1,132	10	28.91	3.73 (2.00, 6.97)
	4	14.254	43,251	56	3.93	1.35 (1.00, 1.83)
R$^{2}$	0.002					
Max. R$^{2}$	0.053					
Log Likelihood	−18,399.580					
Wald Test	1,142.190$$^{***}$$ (df = 22)					
LR Test	1,140.875$$^{***}$$ (df = 22)					
Score Test	1,346.365$$^{***}$$ (df = 22)					

All models adjusted for sex, age group, urban/rural classification, SIMD quintile, ethnicity, and QCovid risk groups

*PY* Person years, *HR* Hazard ratio, *CI* Confidence interval

$$^{*}$$*p*<0.1; $$^{**}$$*p*<0.05; $$^{***}$$*p*<0.01

Among individuals aged 16–74 (Table 1), the fully vaccinated group had a hospitalisation rate of 1.58 per 1,000 person-years (PY), serving as the reference category. Compared to them, those with one missed dose (sub-optimal level 1) had a lower crude hospitalisation rate (1.36 per 1,000 PY) but exhibited a significantly increased hazard ratio (HR: 1.79, 95% CI: 1.54–2.08), suggesting that once covariates were adjusted for, even a single missed dose was associated with a higher risk. The risk increased further with higher levels of undervaccination: HRs were 2.63 (95% CI: 2.04–3.39) for level 2 and 2.34 (95% CI: 2.02–2.72) for level 3. Furthermore, the test statistics (Wald, likelihood ratio, and score tests) all indicate strong overall model significance ($p<0.01$).

In the 75+ age group (Table 2), hospitalisation rates were markedly higher across all groups, reflecting increased vulnerability in older adults. The fully vaccinated had a hospitalisation rate of 7.85 per 1,000 PY. Individuals with a sub-optimal level of one dose had a substantially higher rate (24.42 per 1,000 PY) and a hazard ratio of 2.71 (95% CI: 2.37–3.09). The risk increased with higher undervaccination levels, reaching an HR of 3.92 (95% CI: 3.07–5.00) for sub-optimal level 2 and 3.73 (95% CI: 2.00–6.97) for level 3. Interestingly, those in sub-optimal level 4, a group with distinct vaccination patterns, had a lower rate of hospitalisation (3.93 per 1,000 PY) and a hazard ratio of 1.35 (95% CI: 1.00–1.83), suggesting potential heterogeneity in this subgroup. The comparatively lower hospitalisation risk observed among individuals aged 75+ with undervaccination level 4 could be due to competing risks, whereby more frail individuals are more likely to experience non-hospitalised death (for example, in care homes or community settings).

We now shift our focus from hospitalisation to more severe outcomes, specifically the risk of ICU admission and death.

Table 3 presents the results from the Cox model estimating the risk of ICU admission among individuals aged 16 to 74 years. We observe a clear trend: higher undervaccination levels were associated with increased hazards of ICU admission. Compared to those with no undervaccination, individuals at level 3 exhibit a significantly higher risk (HR = 3.47, 95% CI: 1.85–6.51). Although the absolute event rates remain low (ranging from 0.06 to 0.10 per 1,000 person-years), the hazard ratios suggest a dose–response relationship.

**Table 3:** Cox model of risk of being ICU admitted, 16–74 years old

Variable	Levels	PY (thousands)	Persons	Events	Rate (/1000 PY)	HR (95% CI)
undervaccination	0	945.433	3,105,040	63	0.07	Ref
	1	156.704	502,379	9	0.06	1.43 (0.64, 3.20)
	2	36.397	116,815	<5	0.08	2.80 (0.85, 9.23)
	3	132.011	401,421	13	0.10	3.47 (1.85, 6.51)
R$^{2}$	0.0001					
Max. R$^{2}$	0.001					
Log Likelihood	−1,187.451					
Wald Test	237.980$$^{***}$$ (df = 30)					
LR Test	231.366$$^{***}$$ (df = 30)					
Score Test	770.878$$^{***}$$ (df = 30)					

All models adjusted for sex, age group, urban/rural classification, SIMD quintile, ethnicity, and QCovid risk groups

*PY* Person years, *HR* Hazard ratio, *CI* Confidence interval

$$^{*}$$*p*<0.1; $$^{**}$$*p*<0.05; $$^{***}$$*p*<0.01

In the 75+ age group (Table 4), the association between undervaccination and ICU admission becomes more pronounced. Individuals at sub-optimal level 2 show a markedly increased hazard of ICU admission (HR = 12.53, 95% CI: 3.62–43.44). However, confidence intervals widened at higher sub-optimal levels due to small sample sizes and low event counts. Notably, no ICU admissions were recorded at level 3 in this age group.

**Table 4:** Cox model of risk of being ICU admitted, 75+ years old

Variable	Levels	PY (thousands)	Persons	Events	Rate (/1000 PY)	HR (95% CI)
undervaccination	0	131.121	590,986	16	0.12	Ref
	1	12.085	53,662	5	0.41	3.61 (1.31, 9.95)
	2	2.256	7,351	<5	1.33	12.53 (3.62, 43.44)
	3	0.347	1,132	0	0.00	0 (0, Inf)
	4	14.263	43,251	<5	0.28	6.16 (1.61, 23.52)
R$^{2}$	0.00004					
Max. R$^{2}$	0.001					
Log Likelihood	−351.782					
Wald Test	33.230$^{*}$ (df = 22)					
LR Test	29.056 (df = 22)					
Score Test	40.766$$^{***}$$ (df = 22)					

All models adjusted for sex, age group, urban/rural classification, SIMD quintile, ethnicity, and QCovid risk groups

*PY* Person years, *HR* Hazard ratio, *CI* Confidence interval

$$^{*}$$*p*<0.1; $$^{**}$$*p*<0.05; $$^{***}$$*p*<0.01

Finally, Tables 5 and 6 show the results for the risk of death across age groups. Among individuals aged 16 to 74, the association between undervaccination and mortality was less consistent. While the hazard ratio was elevated at level 3 (HR = 1.53, 95% CI: 0.85–2.77), this result was not statistically significant, and lower levels of undervaccination did not appear associated with increased risk of death. The wide confidence intervals reflected the relatively small number of events in this group.

**Table 5:** Cox model of risk of death, 16–74 years old

Variable	Levels	PY (thousands)	Persons	Events	Rate (/1000 PY)	HR (95% CI)
undervaccination	0	945.441	3,105,041	107	0.11	Ref
	1	156.705	502,379	9	0.06	0.80 (0.40, 1.59)
	2	36.398	116,815	<5	0.03	0.37 (0.05, 2.67)
	3	132.013	401,421	13	0.10	1.53 (0.85, 2.77)
R$^{2}$	0.0001					
Max. R$^{2}$	0.001					
Log Likelihood	−1,777.321					
Wald Test	352.830$$^{***}$$ (df = 19)					
LR Test	324.498$$^{***}$$ (df = 19)					
Score Test	1,085.677$$^{***}$$ (df = 19)					

All models adjusted for sex, age group, urban/rural classification, SIMD quintile, ethnicity, and QCovid risk groups

*PY* Person years, *HR* Hazard ratio, *CI* Confidence interval

$$^{*}$$*p*<0.1; $$^{**}$$*p*<0.05; $$^{***}$$*p*<0.01

**Table 6:** Cox model of risk of death, 75+ years old

Variable	Levels	PY (thousands)	Persons	Events	Rate (/1000 PY)	HR (95% CI)
undervaccination	0	131.123	590,990	242	1.85	Ref
	1	12.086	53,662	97	8.03	3.94 (3.10, 5.00)
	2	2.256	7,351	21	9.31	5.23 (3.34, 8.19)
	3	0.347	1,132	<5	11.52	6.63 (2.46, 17.83)
	4	14.263	43,251	17	1.19	1.48 (0.84, 2.63)
R$^{2}$	0.001					
Max. R$^{2}$	0.014					
Log Likelihood	−4,702.530					
Wald Test	475.570$$^{***}$$ (df = 22)					
LR Test	485.865$$^{***}$$ (df = 22)					
Score Test	586.484$$^{***}$$ (df = 22)					

All models adjusted for sex, age group, urban/rural classification, SIMD quintile, ethnicity, and QCovid risk groups

*PY* Person years, *HR* Hazard ratio, *CI* Confidence interval

$$^{*}$$*p*<0.1; $$^{**}$$*p*<0.05; $$^{***}$$*p*<0.01

In contrast, the 75+ group displays a stronger and clearer gradient in mortality risk with increasing undervaccination (Table 6). Individuals with sub-optimal level 1 already show a substantially higher risk of death compared to those fully vaccinated (HR = 3.94, 95% CI: 3.10–5.00). This risk increases further at level 2 (HR = 5.23, 95% CI: 3.34–8.19), and peaks at level 3 (HR = 6.63, 95% CI: 2.46–17.83), although the number of events was very small in this group. Interestingly, the association appeared to weaken at level 4 (HR = 1.48, 95% CI: 0.84–2.63), suggesting a possible non-linear relationship or increased heterogeneity at the highest sub-optimal level. Despite the overall low event rates in the cohort, these results support the presence of a dose–response relationship between undervaccination and mortality risk among older adults.

Beyond individual-level clinical and economic outcomes, it was also important to estimate the potential system-wide costs associated with sub-optimal COVID-19 vaccine uptake. Table S4 in the Supplementary Materials presents the projected costs of vaccinating undervaccinated populations across two age groups in Scotland as of Summer 2022, based on observed undervaccination. For each age group, we provide the number of individuals at each sub-optimal level (0 to 4), the doses required to achieve full coverage, and the associated costs of both full and partial vaccination using two pricing structures (Pfizer and Moderna).

These estimates illustrate that a relatively small proportion of the population accounts for a disproportionate share of undervaccination —and therefore potential savings. For instance, among those aged 16–74, only 114,946 individuals had a sub-optimal of two doses, yet this contributes to a much larger aggregate cost given the total of 1.3 million individuals undervaccinated in this group. Full vaccination of all 16–74 year olds with a sub-optimal level of vaccination would require 2.76 million doses and incur an estimated cost of £197.4 million using Pfizer prices, or £205.7 million using Moderna. In contrast, for the 75+ group, full vaccination requires fewer doses overall (277,305), but the costs per dose and the higher risk profile of this population make these investments particularly cost-effective from a health system perspective.

Importantly, the distribution across sub-optimal vaccination levels shows that the number of individuals at the highest deficit categories remains relatively small. For example, only 1,096 individuals aged 75+ had a undervaccination of three, and 51,699 had a sub-optimal of four, yet both groups remain vulnerable and represent potential high-impact targets for future booster campaigns.

While these projections highlight the investment needed for complete vaccine coverage, it is equally crucial to quantify the actual financial burden incurred due to severe COVID-19 outcomes in the undervaccinated population. Table 7 presents a combined analysis of hospitalisation costs, ICU costs, and deaths, offering a financial perspective on the impact of COVID-19. This analysis complements the earlier findings on hospitalisation risk, ICU admission, and mortality by quantifying the economic burden associated with these outcomes. For hospitalisation costs, the data revealed a substantial economic burden, particularly among older adults (75+ years), where the actual costs reached £4,715,675, compared to £2,647,888 for the 16–74 age group. The savings achieved through reduced hospitalisations were also considerably higher in the 75+ group (£1,411,647) than in the younger group (£494,926.70), underscoring the greater economic impact of hospitalisations in older populations, which aligns with the higher hospitalisation rates and hazard ratios observed in this age group in Tables 1 and 2. ICU costs, while lower in absolute terms than overall hospitalisation costs, still represented a significant expense, with actual costs of £246,486.30 for the 16–74 age group and £70,488.53 for the 75+ group. These figures correspond to the findings in Tables 3 and 4, which highlighted the increased risk of ICU admission with higher undervaccination levels, especially in older adults, thus translating to higher potential costs. The data on deaths, although not directly associated with costs in Table S4 in the Supplementary Materials, provides context for the potential economic impact averted through vaccination by preventing these severe outcomes. The earlier analysis in Tables 5, 6 and 7 demonstrated a clear dose-response relationship between undervaccination and mortality risk, particularly in the 75+ age group, where higher undervaccination levels were associated with significantly increased hazard ratios for death. Integrating these cost findings with the previous observations on hospitalisation, ICU admission, and mortality risk reinforces the conclusion that undervaccination not only increases the risk of severe health outcomes but also imposes a substantial economic burden on the healthcare system, especially concerning older adults. While the base-case economic analysis includes QALY gains from averted mortality only, integrated sensitivity analyses incorporating morbidity-related quality-of-life losses (Supplementary Materials, Section 2–4) indicate that inclusion of hospitalisation- and ICU-related utility decrements increases total QALYs gained by approximately 14% over the study period. This suggests that the mortality-only base case represents a conservative lower-bound estimate of vaccination benefits.

**Table 7:** Combined analysis of hospitalisation costs, ICU costs, and deaths

Age	Actual events	Expected events	N. expected var.	Actual duration	Expected duration	Variance duration	Actual cost	Counterfactual cost	Savings
**Hospitalisation Costs**									
16–74	1998	1727.589	19.280	12260.5	10110.561	82.485	2647888.79	2152962.09	494926.70
75+	1458	1192.462	45.601	21616.0	15617.333	30078.167	4715675.46	3304027.88	1411647.58
**ICU Costs**									
16–74	88	72.713	37.273	415.25	391.380	14682.286	246486.30	228568.95	17917.35
75+	28	18.274	519.320	103.25	66.710	3809.584	70488.53	49740.35	20748.18
**Deaths**									
16–74	130	127.453	4.695	9.738	1.023	-	-	-	-
75+	381	280.713	124.819	629.075	177.448	-	-	-	-

Our findings show that unvaccinated individuals incurred significantly higher rates of severe COVID-19 outcomes and associated healthcare costs. On average, unvaccinated patients generated over five times the cost of their fully vaccinated counterparts, primarily due to increased hospital and ICU admissions. These differences were particularly pronounced in older age groups. In a counterfactual scenario assuming full vaccination, we estimate substantial reductions in hospitalisations, ICU stays, and NHS costs across the study period.

## Discussion and conclusions

This study provides robust empirical evidence on the economic consequences of COVID-19 undervaccination in Scotland, using comprehensive national-level data from the EAVE II cohort. Our primary contribution was a novel empirical evaluation of the real-world, patient-level cost differentials associated with vaccination status within a publicly funded healthcare system. By quantifying the increased healthcare costs associated with hospitalisations, ICU admissions, and mortality among undervaccinated individuals, our findings reinforce the value of full vaccine uptake as both a clinical and economic imperative. Our methodology complements the work of Kerr et al. [19] by shifting the focus from averted outcomes to the direct economic costs incurred. In doing so, our research directly quantifies the substantial additional NHS costs incurred by undervaccinated individuals, framing our findings as a clear economic imperative for continued investment in immunization programs, rather than a marginal expenditure.

Our analysis reveals that unvaccinated patients incurred, on average, more than five times the healthcare costs of vaccinated patients, primarily due to greater utilisation of hospital and ICU services. These disparities persisted across age groups and were especially marked during periods of heightened system demand. This study offers a novel contribution by presenting one of the first empirical evaluations of real-world, patient-level cost differentials associated with COVID-19 vaccination in a UK context, using administrative health data from NHS Scotland. By quantifying the direct cost savings associated with vaccination, our findings inform future public health policy and reinforce the value of sustained investment in immunisation programmes as a key component of pandemic preparedness and NHS resilience.

A clear dose–response relationship was observed between undervaccination and adverse outcomes, particularly among individuals 75 years and older. Each additional missed dose was associated with significantly elevated hazard ratios for hospitalisation, ICU admission, and death. These clinical outcomes translated into substantial additional NHS costs. Notably, the estimated savings from full vaccination in this age group exceeded £1.4 million in hospitalisation costs alone during the summer 2022 study period. Similar trends, though less pronounced, were observed in the 16–74 age group, underlining the broader relevance of vaccination for working-age adults.

These results align with international evidence demonstrating the cost-effectiveness of COVID-19 vaccination programmes. However, our study contributes new UK-specific data based on real-world outcomes, addressing a critical evidence gap in the context of a publicly funded healthcare system. Furthermore, by incorporating a counterfactual analysis, we provide estimates of the cost burden potentially averted through improved vaccine coverage, adding practical relevance for policymakers.

Health inequalities remain a significant concern. Lower vaccination rates among ethnic minority groups, socioeconomically deprived populations, and younger individuals not only perpetuate disparities in COVID-19 outcomes but also lead to disproportionate economic burdens. Targeted efforts to increase vaccine uptake in these communities were therefore warranted, not only as a matter of health equity but also for fiscal sustainability.

Our study is subject to several limitations. First, certain findings, particularly regarding ICU admission and mortality among younger cohorts, were constrained by low event counts. This reduced statistical power and resulted in wider confidence intervals for these specific subgroups. Second, the non-monotonic hazard ratios observed at higher undervaccination levels in the 75+ age group may reflect competing risks. For instance, frailer individuals might be more likely to die in community or care-home settings without hospital admission, potentially masking the true relationship between vaccination status and disease severity.

Third, while we adjusted for a wide range of demographic and clinical factors, BMI was excluded due to high missingness. Since GPs often record BMI selectively such as for patients outside the healthy range, we did not believe a missing at random assumption was appropriate. However omitting BMI may leave some residual confounding. More broadly, as this is an observational study, residual confounding from unobservable factors, including occupational exposure, frailty, or health-seeking behavior, cannot be entirely ruled out.

Fourth, our counterfactual analysis relies on the assumption of conditional exchangeability. Under this framework, the differences between observed and hypothetical scenarios should be interpreted as policy-relevant associations rather than definitive causal effects. Accordingly, our estimates represent the potential scale of avertable burden under full vaccination coverage rather than precise causal impacts of vaccination itself.

Finally, our economic analysis provides conservative lower-bound estimates. By design, we captured only direct NHS hospital and ICU costs, excluding primary care, emergency services, long COVID care, social services, and indirect costs such as productivity losses. Regarding health utility, our primary QALY estimates are based solely on averted mortality; however, as shown in our sensitivity analysis in the Supplementary Materials, incorporating morbidity-related quality-of-life losses increases the projected health benefits of vaccination.

Nevertheless, this analysis offers timely insights into the preventable costs associated with undervaccination during a period of ongoing viral transmission and NHS strain. As the UK continues to navigate future waves of SARS-CoV-2 and other respiratory pathogens, ensuring sustained and equitable vaccine coverage remains a cornerstone of pandemic preparedness and health system resilience.

In conclusion, this study provides a robust, real-world assessment of the epidemiological and economic consequences of COVID-19 undervaccination in a publicly funded healthcare system. Using comprehensive population-level data from Scotland, we show that undervaccination is associated with substantially higher risks of severe outcomes and markedly higher direct NHS costs. While our findings are subject to the assumptions inherent in observational and counterfactual analyses, they highlight the scale of potentially avertable healthcare burden associated with sub-optimal vaccine uptake and reinforce the importance of sustained and equitable vaccination strategies for health system resilience.

## Supplementary Information

Supplementary Material 1.

## Data Availability

The data utilized in this study consist of sensitive, individual-level, linked health records from the Early Pandemic Evaluation and Enhanced Surveillance of COVID-19 (EAVE II) platform in Scotland. Due to the highly confidential nature of these data and to protect patient privacy, they are not publicly available. Access to the EAVE II platform is strictly controlled and granted only to approved researchers operating within secure trusted research environments in accordance with stringent ethical and data governance protocols. Researchers interested in accessing similar data for future studies may submit a formal application to Public Health Scotland, following their established data access procedures and subject to ethical approval. This ensures adherence to national guidelines for the use of health data for research purposes.
