# Supplementary material for: The economic burden of COVID-19 undervaccination: costs of hospitalisation, ICU admission, and death in Scotland
Source: Health Econ Rev. 2026 Apr 21;16:69. doi: 10.1186/s13561-026-00775-3 (PMC13227698; doi:10.1186/s13561-026-00775-3)
Supplement: Supplementary file 1 — Supplementary Material 1. [file 13561_2026_775_MOESM1_ESM.pdf]

# Supplemental Materials

## 1 Supplemental Tables and Figures

Table S1: Data Source

| Nation | Population | Data Platform | Key Data Sources |
|---|---|---|---|
| Scotland | 5.4 million | EAVE II | • Vaccination data<br>• PCR testing data<br>• LFT data<br>• **Primary care data**: GP records including QCovid risk groups<br>• Hospital admission data<br>• Mortality data<br>• Demographic data<br>• **Other healthcare data**: NHS24 calls, A&E, outpatients, maternity services, etc. |

Table S2: Scotland Vaccination Requirements for Population Aged 16+ (Prior to 1 June 2022)

| Age Group | Doses Eligible For | Last Dose Offered | Sub-optimal Vaccination Status |
|---|---|---|---|
| 16–74 | 2 vaccine + 1 booster doses | 28/02/2022 | Unvaccinated, 1 or 2 doses |
| 75+ | 2 vaccine + 2 booster doses | 25/03/2022 | Unvaccinated, 1, 2 or 3 doses |

Table S3: Data Description

| Cohort Description | Fully-vaccinated | Undervaccinated |
|---|---|---|
| **Total** | 3282712 (65.8) | 1709786 (34.2) |
| **Sex** | | |
| Female | 1734510 (69.0) | 780972 (31.0) |
| Male | 1548202 (62.5) | 928814 (37.5) |
| **Age** | | |
| Mean (SD) | 51.1 (19.9) | 30.8 (19.0) |
| **Age group** | | |
| 5-11 | 78601 (20.4) | 306239 (79.6) |
| 12-15 | 108795 (46.9) | 123103 (53.1) |
| 16-17 | 18257 (17.0) | 88850 (83.0) |
| 18-24 | 190540 (50.4) | 187288 (49.6) |
| 25-29 | 153264 (45.6) | 183160 (54.4) |
| 30-34 | 182094 (49.7) | 184350 (50.3) |
| 35-39 | 207025 (56.8) | 157143 (43.2) |
| 40-44 | 225466 (64.7) | 122816 (35.3) |
| 45-49 | 242655 (72.7) | 91012 (27.3) |
| 50-54 | 311074 (81.2) | 71811 (18.8) |
| 55-59 | 337579 (86.4) | 53338 (13.6) |
| 60-64 | 318328 (90.0) | 35290 (10.0) |
| 65-69 | 275447 (92.7) | 21782 (7.3) |
| 70-74 | 254264 (94.7) | 14206 (5.3) |
| 75-79 | 172477 (85.2) | 29957 (14.8) |
| 80-84 | 111959 (85.2) | 19379 (14.8) |
| 85+ | 94887 (82.5) | 20062 (17.5) |
| **SIMD quintile** | | |
| 1 - Most deprived | 548104 (53.9) | 469291 (46.1) |
| 2 | 617913 (62.4) | 371911 (37.6) |
| 3 | 660294 (68.1) | 309956 (31.9) |
| 4 | 711679 (71.9) | 277580 (28.1) |
| 5 - Least deprived | 726385 (73.6) | 260468 (26.4) |
| (Missing) | 18337 (47.1) | 20580 (52.9) |
| **Urban/rural classification** | | |
| Urban | 2271393 (63.7) | 1292039 (36.3) |
| Rural | 992982 (71.4) | 397167 (28.6) |
| (Missing) | 18337 (47.1) | 20580 (52.9) |
| **Ethnicity** | | |
| White | 2294643 (69.7) | 997853 (30.3) |

| | | |
|---|---|---|
| Asian | 70667 (56.8) | 53737 (43.2) |
| Black | 12314 (39.0) | 19222 (61.0) |
| Mixed | 14174 (43.7) | 18226 (56.3) |
| Other | 11928 (42.5) | 16146 (57.5) |
| Unknown | 878986 (59.2) | 604602 (40.8) |
| **Number of people in household** | | |
| 3-5 | 1237080 (60.2) | 818748 (39.8) |
| 1 | 938914 (69.3) | 416596 (30.7) |
| 2 | 1009904 (75.2) | 332646 (24.8) |
| 6-10 | 82881 (39.1) | 128884 (60.9) |
| 11+ | 13933 (51.9) | 12912 (48.1) |
| **Mean household age** | | |
| Mean (SD) | 46.9 (19.0) | 31.7 (15.5) |
| **Number of risk groups** | | |
| 0 | 2153166 (60.4) | 1411655 (39.6) |
| 1 | 770490 (76.5) | 237198 (23.5) |
| 2 | 232639 (85.3) | 40213 (14.7) |
| 3 | 82359 (86.5) | 12857 (13.5) |
| 4 | 30005 (85.5) | 5088 (14.5) |
| 5+ | 14053 (83.5) | 2775 (16.5) |
| **Last positive test** | | |
| never_positive | 2508548 (66.8) | 1247646 (33.2) |
| 0-13_weeks | 140142 (67.4) | 67905 (32.6) |
| 14-26_weeks | 291218 (61.3) | 184199 (38.7) |
| 27+_weeks | 342804 (62.0) | 210036 (38.0) |
| **Housing category** | | |
| Neither | 3264310 (65.7) | 1707279 (34.3) |
| Care home | 18170 (89.4) | 2148 (10.6) |
| Homeless | 232 (39.3) | 359 (60.7) |
| **Learning disability/Down's syndrome** | | |
| Neither | 3239993 (65.8) | 1682581 (34.2) |
| Down's syndrome | 1737 (80.2) | 428 (19.8) |
| Learning disability | 40982 (60.5) | 26777 (39.5) |
| **Chronic Kidney Disease** | | |
| No CKD | 3145643 (65.0) | 1690259 (35.0) |
| CKD 3 | 137050 (87.5) | 19524 (12.5) |
| CKD 4 | 15 (83.3) | 3 (16.7) |
| CKD 5 | 4 (100.0) | 0 (0.0) |
| **Atrial Fibrillation** | 93533 (87.7) | 13134 (12.3) |
| **Asthma** | 374458 (72.0) | 145793 (28.0) |

| | | |
|---|---|---|
| **Blood cancer** | 16812 (86.9) | 2530 (13.1) |
| **Congestive Cardiac Failure** | 37130 (85.9) | 6112 (14.1) |
| **Cerebral Palsy** | 5075 (77.5) | 1472 (22.5) |
| **Coronary heart disease** | 158516 (87.5) | 22620 (12.5) |
| **Liver cirrhosis** | 8610 (85.0) | 1515 (15.0) |
| **Congenital heart disease** | 16911 (71.1) | 6881 (28.9) |
| **COPD** | 94666 (84.3) | 17586 (15.7) |
| **Dementia** | 16453 (83.4) | 3277 (16.6) |
| **Diabetes Type 1** | 50579 (76.2) | 15775 (23.8) |
| **Diabetes Type 2** | 157793 (74.3) | 54574 (25.7) |
| **Epilepsy** | 2760 (74.1) | 966 (25.9) |
| **Hip, wrist, spine, humerus fracture** | 1442 (80.2) | 357 (19.8) |
| **HIV/AIDS** | 15876 (88.1) | 2150 (11.9) |
| **Severe combine immunodeficiency** | 7814 (88.3) | 1033 (11.7) |
| **Neurological conditions** | 5529 (85.5) | 934 (14.5) |
| **Parkinson's** | 19880 (87.3) | 2887 (12.7) |
| **Pulmonary hypertension** | 9469 (84.8) | 1691 (15.2) |
| **Cystic fibrosis, bronchiectasis or alveolitis** | 73474 (85.9) | 12070 (14.1) |
| **Peripheral vascular disease** | 4303 (87.6) | 609 (12.4) |
| **SLE or rheumatoid arthritis** | 13657 (73.2) | 5006 (26.8) |
| **Lung, oral cancer** | 140 (51.3) | 133 (48.7) |
| **Severe mental illness** | 93549 (86.0) | 15233 (14.0) |
| **Sickle cell disease Imm Def** | 33026 (83.5) | 6532 (16.5) |
| **Stroke, transient ischaemic attack** | 17744 (85.3) | 3047 (14.7) |
| **Venous thromboembolism** | 212065 (88.3) | 28044 (11.7) |
| **Smoking status** | | |
| Non-smoker | 1509903 (74.0) | 529957 (26.0) |
| Ex-smoker | 506000 (81.6) | 113905 (18.4) |
| Smoker | 730015 (70.1) | 310999 (29.9) |
| (Missing) | 536794 (41.6) | 754925 (58.4) |
| **Blood pressure** | | |
| Normal | 2098491 (76.3) | 653569 (23.7) |
| Low | 46706 (64.3) | 25975 (35.7) |
| High | 381373 (83.3) | 76543 (16.7) |
| Very high | 76808 (81.4) | 17588 (18.6) |
| (Missing) | 679334 (42.1) | 936111 (57.9) |

Table S4: Vaccination Costs by Age Group and undervaccination

| Age | Vaccine sub-optimal | $n$ | Doses Required | Undervax. Pop. | Full Cost Pfizer | Partial Cost Pfizer | Full Cost Moderna | Partial Cost Moderna |
|---|---|---|---|---|---|---|---|---|
| 16–74 | 0 | 2,832,992 | 2,759,200 | 1,287,186 | 197,393,168 | 92,085,286 | 205,670,768 | 95,946,844 |
| 16–74 | 1 | 493,706 | 2,759,200 | 1,287,186 | 197,393,168 | 92,085,286 | 205,670,768 | 95,946,844 |
| 16–74 | 2 | 114,946 | 2,759,200 | 1,287,186 | 197,393,168 | 92,085,286 | 205,670,768 | 95,946,844 |
| 16–74 | 3 | 678,534 | 2,759,200 | 1,287,186 | 197,393,168 | 92,085,286 | 205,670,768 | 95,946,844 |
| 75+ | 0 | 384,916 | 277,305 | 112,711 | 19,838,400 | 8,063,345 | 20,670,315 | 8,401,478 |
| 75+ | 1 | 52,611 | 277,305 | 112,711 | 19,838,400 | 8,063,345 | 20,670,315 | 8,401,478 |
| 75+ | 2 | 7,305 | 277,305 | 112,711 | 19,838,400 | 8,063,345 | 20,670,315 | 8,401,478 |
| 75+ | 3 | 1,096 | 277,305 | 112,711 | 19,838,400 | 8,063,345 | 20,670,315 | 8,401,478 |
| 75+ | 4 | 51,699 | 277,305 | 112,711 | 19,838,400 | 8,063,345 | 20,670,315 | 8,401,478 |

*Note*: "Cost of partial vaccination" means the cost of giving one dose to everyone who was undervaccinated. "Cost of full vaccination" means having given as many doses as would have been required for that person to be fully vaccinated. With the exception of the columns "undervaccination " and "$n$", every other column has numbers repeated three times.

# 2 Morbidity-Related QALY Losses: Literature Review and Quantitative Illustration

## 2.1 Understanding the Current Approach

As noted in our manuscript (Section 4.5.1, page 15), we currently calculate QALYs gained exclusively from averted deaths, following the methodology of Kohli et al. This approach applies age-specific utility weights and a 3.5% discount rate to estimate the quality-equivalent cost of death (QECD). However, as the reviewer correctly identifies, this omits quality-of-life decrements experienced during acute illness episodes (hospitalisation and ICU admission).

## 2.2 UK-Specific Utility Values for COVID-19 Health States

To address this limitation, we conducted a focused literature review to identify UK-specific utility values for COVID-19 morbidity states. We identified a highly relevant vignette study presented at ISPOR Europe 2023, which estimated utility values using a representative UK population sample ($N = 500$).

**Study Details:**

- **Reference:** Ntais D, Ntim V, Barton S, et al. Health-Related Quality of Life in COVID-19: A Vignette Study. Presented at ISPOR Annual European Congress, Copenhagen, Denmark, November 12-15, 2023.
- **Method:** Eight health state vignettes covering COVID-19 severity spectrum, valued using EQ-5D-5L questionnaire completed by UK general population as patient proxies
- **Population:** Representative UK adults (mean age 44.15 years, 48.8% male, 80.4% White, 83.8% vaccinated)
- **Conversion:** EQ-5D-5L crosswalk algorithm used to estimate EQ-5D-3L utility scores per NICE recommendations

## 2.3 Calculating Utility Decrements

To estimate QALY losses from morbidity, we calculate the utility decrement as the difference between baseline health status and the acute disease state:

$$\text{Utility Decrement} = \text{Baseline Utility} - \text{Disease State Utility} \tag{1}$$

For general hospital ward:

$$\text{Decrement} = 0.73 - (-0.19) = 0.92 \text{ QALYs per year} \tag{2}$$

For ICU admission:

$$\text{Decrement} = 0.73 - (-0.37) = 1.10 \text{ QALYs per year} \tag{3}$$

Table S5: UK Population-Derived Utility Values for COVID-19 Health States

| Health State | Utility Value (Mean) | Description |
|---|---|---|
| Baseline (pre-infection) | 0.73 | No COVID-19, underlying condition present |
| General hospital ward (Severe) | −0.19 | Oxygen via nasal cannula, symptoms include fever, cough, fatigue, confusion, muscle pain |
| High Dependency Unit (HDU) | −0.09 | Oxygen via face mask, symptoms as above |
| Intensive Care Unit (Critical) | −0.37 | Intubated, cannot breathe independently, will die without treatment |

*Note: Negative utility values indicate health states perceived as worse than death by the general population. Values highlighted represent the primary inputs for our sensitivity analysis.*

**Scaling by days:**

For general hospital ward (7 days stay):

$$\text{QALY loss} = 0.92 \times \frac{7}{365} \approx 0.018 \text{ QALYs ( 6.5 quality-adjusted life days)} \tag{4}$$

For ICU admission (10 days stay):

$$\text{QALY loss} = 1.10 \times \frac{10}{365} \approx 0.030 \text{ QALYs (11 quality-adjusted life days)} \tag{5}$$

These decrements quantify the substantial reduction in health-related quality of life experienced during acute COVID-19 requiring hospital or ICU care.

## 2.4 Quantitative Illustration: Morbidity QALY Losses in Our Cohort

Using actual hospitalisation and ICU durations from our study (Table 8 of manuscript), we can estimate the morbidity-related QALY losses that would complement our mortality-based QALY calculations:

Table S6: Observed Duration of Hospital and ICU Stays (Study Period: June-September 2022)

| Age Group | Hospital Duration (days) | ICU Duration (days) |
|---|---|---|
| 16-74 years | 12,260.5 | 415.25 |
| 75+ years | 21,616.0 | 103.25 |

*Source: Manuscript Table 8*

**Calculation Methodology:**

$$\text{QALY Loss} = \text{Utility Decrement} \times \left(\frac{\text{Duration in days}}{\text{365 days/year}}\right) \tag{6}$$

Table S7: Estimated Morbidity-Related QALY Losses

| Age Group | Hospital QALY Loss | ICU QALY Loss | Total Morbidity QALYs Lost |
|---|---|---|---|
| 16-74 years | 30.90 | 1.25 | 32.15 |
| 75+ years | 54.48 | 0.31 | 54.79 |
| **Total** | **85.38** | **1.56** | **86.94** |

*Calculations: Age 16-74: Hospital* $= 0.92 \times (12,260.5 \div 365) = 30.90$*; ICU* $= 1.10 \times (415.25 \div 365) = 1.25$. *Age 75+: Hospital* $= 0.92 \times (21,616.0 \div 365) = 54.48$*; ICU* $= 1.10 \times (103.25 \div 365) = 0.31$.

## 2.5 Interpretation and Implications

These estimates demonstrate that incorporating morbidity-related QALY losses would substantially increase the measured health benefits of vaccination:

- **Aggregate morbidity burden:** Approximately 87 QALYs were lost due to morbidity from hospitalisation and ICU stays during our 4-month study period (June-September 2022).
- **Age-specific patterns:** Older adults (75+) experienced higher absolute morbidity QALY losses (54.79 vs 32.15), reflecting both longer hospital stays and higher vulnerability.
- **ICU contribution:** Despite shorter durations, ICU stays involved more severe utility decrements (1.10 vs 0.92 per year), contributing meaningfully to overall morbidity burden.
- **Conservative baseline:** Our current mortality-only QALY estimates therefore represent a lower bound. Including morbidity would strengthen our conclusions about vaccination cost-effectiveness.

## 3 Sensitivity Analysis Framework

Based on our literature review, we employed the following sensitivity analysis as robustness check:

Table S8: Proposed Sensitivity Analysis for Utility Weights

| Scenario | Interpretation | Hospital Ward Utility | ICU Utility | Source |
|---|---|---|---|---|
| Base case (current) | Mortality-only QALYs (no morbidity) | N/A (mortality only) | N/A (mortality only) | Kohli et al. |
| Lower bound | Mild short-term disutility | 0.63 (10% decrement = −0.10) | 0.45 (28% decrement = −0.28) | PLOS One 2021 (UK Long COVID Model) |
| Upper bound | Acute illness valued as very poor health | −0.19 | −0.37 | ISPOR 2023 (UK Vignette Study) |

*Note: All values are EQ-5D-3L utility scores. Baseline utility = 0.73 for population with underlying conditions. The base case excludes morbidity by design; the lower- and upper-bound scenarios do not represent statistical uncertainty but alternative, literature-based valuations of acute COVID-19 morbidity severity.*

This framework would demonstrate that our findings are robust across a range of plausible utility assumptions, with the upper bound scenario (ISPOR values) showing the largest potential health benefits from vaccination.

# 4 Integrated Sensitivity Analysis Including Morbidity QALYs

## 4.1 Mortality-Related QALYs (Base Case)

From the observed–expected counterfactual analysis (Table **??** of the main manuscript), the estimated number of deaths potentially averted under full vaccination was:

- 2.55 deaths in individuals aged 16–74
- 100.29 deaths in individuals aged 75+

This corresponds to a total of approximately 102.84 deaths averted during the four-month study period.

Following the approach of Kohli et al., mortality-related QALYs were calculated using age-specific discounted remaining life expectancy (3.5% annual discount rate) and age-specific utility weights. Using conservative approximations consistent with UK life expectancy patterns:

- Ages 16–74: $\sim 15$ discounted QALYs per death
- Ages 75+: $\sim 6$ discounted QALYs per death

This yields:

$$\text{Mortality QALYs}_{16--74} = 2.55 \times 15 = 38.2 \tag{7}$$

$$\text{Mortality QALYs}_{75+} = 100.29 \times 6 = 601.7 \tag{8}$$

$$\textbf{Total Mortality QALYs} \approx 640 \tag{9}$$

## 4.2 Morbidity-Related QALYs

As estimated in Section S7, morbidity-related QALY losses associated with hospitalisation and ICU admission during the study period totalled:

$$\textbf{Total Morbidity QALYs} = 86.94 \tag{10}$$

## 4.3 Combined QALYs Gained

Adding morbidity to mortality-related QALYs:

$$\textbf{Total QALYs (Mortality + Morbidity)} = 640 + 86.94 = 726.94 \tag{11}$$

Thus, inclusion of short-term morbidity increases total QALYs gained by:

$$\frac{86.94}{640} \approx 13.6\% \tag{12}$$

### 4.4 Implications for Cost-Effectiveness

From Table **??**, total NHS savings from reduced hospital and ICU utilisation were approximately £1.95 million over the study period.

If $C$ denotes the incremental net cost of vaccination (vaccination costs minus healthcare savings), then:

$$ICER_{\text{mortality-only}} = \frac{C}{640} \tag{13}$$

$$ICER_{\text{mortality+morbidity}} = \frac{C}{727} \tag{14}$$

Inclusion of morbidity therefore reduces the ICER by approximately 12–14%, improving the cost-effectiveness profile of vaccination.

### 4.5 Interpretation

This integrated sensitivity analysis suggests that:

- Inclusion of short-term morbidity increases total QALYs by approximately 14%.
- Cost-effectiveness improves proportionally when morbidity is included.

Given the short four-month time horizon and the exclusion of long COVID, primary care costs, and broader societal impacts, these estimates remain conservative.

## References


[1] Ntais D, Ntim V, Barton S, Porteous A, Ng A, Page J, Puenpatom A, Siegartel L. Health-Related Quality of Life in COVID-19: A Vignette Study. Presented at the ISPOR Annual European Congress, Copenhagen, Denmark, November 12-15, 2023. Poster PCR63.

[2] Briggs A, Vassall A, Geffen N, Miners A, Phillips AN, et al. A model framework for projecting the prevalence and impact of Long-COVID in the UK. *PLOS One.* 2021;16(12):e0260843.

[3] Kohli M, Maschio M, Lee A, Joshi K, Carroll S, Balogh O, van de Velde N, Beck E. The potential clinical impact and cost-effectiveness of the updated COVID-19 mRNA autumn 2024 vaccines in the United Kingdom. *Journal of Medical Economics.* 2024.
